# Improving the Measurement of Maternal Mortality: The Sisterhood Method Revisited

**DOI:** 10.1371/journal.pone.0059834

**Published:** 2013-04-02

**Authors:** Leena Merdad, Kenneth Hill, Wendy Graham

**Affiliations:** 1 Department of Global Health and Population, Harvard School of Public Health, Boston, Massachusetts, United States of America; 2 Harvard Center for Population and Development Studies, Cambridge, Massachusetts, United States of America; 3 Institute of Applied Health Sciences, School of Medicine and Dentistry, University of Aberdeen, Aberdeen, United Kingdom; 4 Department of Preventive Dental Sciences, Faculty of Dentistry, King Abdulaziz University, Jeddah, Saudi Arabia; Indiana University, United States of America

## Abstract

**Background:**

Over the past several decades the efforts to improve maternal survival and the consequent demand for accurate estimates of maternal mortality have increased. However, measuring maternal mortality remains a difficult task especially in developing countries with weak information systems. Sibling histories included in household surveys (most notably the Demographic and Health Surveys (DHS)) have emerged as an important source of maternal mortality data. Data have been mainly collected from women and have not been widely collected from men due to concerns about data quality. We assess data quality of histories obtained from men and the potential to improve the efficiency of surveys measuring maternal mortality by collecting such data.

**Methods and Findings:**

We used data from 10 Demographic and Health Surveys (DHS) that have included a full sibling history in both their women’s and men’s questionnaires. We estimated adult and maternal mortality indicators from histories obtained from men and women. We assessed the completeness and accuracy of these histories using several indicators of data quality. Our study finds that mortality estimates based on sibling histories obtained from men do not systematically or significantly differ from those obtained from women. Quality indicators were similar when comparing data from men and women. Pooling data obtained from men and women produced narrower confidence intervals.

**Conclusion:**

From experience across nine developing countries, sibling history data obtained from men appear to be a reliable source of information on adult and maternal mortality. Given that there are no significant differences between mortality estimates based on data obtained from men and women, data can be pooled to increase efficiency. This finding improves the feasibility for countries to generate robust empirical estimates of adult and maternal mortality from surveys. Further we recommend that male sibling histories be collected from all sample households rather than from a subsample.

## Introduction

An estimated 287,000 women died in 2010 from obstetric causes [1]. Worldwide efforts to improve maternal health have increased in the last several decades [2], [3]. Beginning with the Safe Motherhood Initiative in 1987 several international summits and conferences have emphasized the importance of maternal health and the 2000 UN Millennium Summit declared reducing maternal mortality by three-quarters by 2015 as one of the targets of Millennium Development Goal Five. This increased interest in maternal health has boosted the demand for accurate estimates of maternal mortality [4]. Tracking progress toward the development goals and monitoring and evaluating the effectiveness of sexual and reproductive health programs requires timely maternal mortality estimates [5]. Moreover, the increased international concern with accountability and rational resource allocation is reflected in the recent UN Commission on Information and Accountability report that includes continued monitoring of maternal mortality as a priority [6]. However, measuring maternal mortality is a difficult task [7].

### The Challenge of Measuring Maternal Mortality

A maternal death is defined in the *International statistical classification of disease and related health problems*, tenth revision (ICD-10), as the “death of a woman while pregnant or within 42 days of the termination of a pregnancy, irrespective of the duration and site of the pregnancy, from any cause related to or aggravated by the pregnancy or its management but not from accidental or incidental causes”. Identifying a death as maternal thus requires assigning a specific cause of death. Even in developed countries with complete registration of deaths, maternal deaths are often underreported due to misclassification [8]. In developing countries underreporting of maternal deaths is further compounded by incomplete or non-existent registration of vital events and lack of medical certification of cause of death. This difficulty in identifying maternal deaths has led to a focus on “pregnancy-related death”, defined in ICD-10 as “the death of a woman while pregnant or within 42 days of the termination of pregnancy, irrespective of cause of death”. The identification of pregnancy-related deaths requires information only on the timing of death relative to pregnancy and thus on answers to apparently simple questions; it is for this reason that this definition is used in censuses and surveys instead of attempting to focus on true maternal deaths. Using the time of death to define pregnancy-related deaths, however, means the inclusion of incidental and accidental deaths. It has been suggested that this overestimation is counterbalanced by the under-reporting of pregnancy-related deaths, and thus that reported pregnancy-related deaths might approximate maternal deaths, although this conclusion remains controversial [9]. In what follows, we shall refer to pregnancy-related deaths or mortality as maternal deaths or mortality, as is common practice in the literature.

While civil registration systems that regularly record births and deaths are generally considered the gold standard for mortality data [10], these systems are absent, underdeveloped or incomplete in most developing countries [11]. In the absence of reliable registration data, interim data sources and methods of analysis have been developed to complement civil registration systems and provide estimates of mortality [12]. However, an additional problem facing such methods is that maternal deaths are relatively infrequent (the annual number of maternal deaths is roughly 5% of the number of deaths of children under age 5), thus requiring large samples to achieve statistical precision. These interim sources of maternal mortality data include sample vital registration with verbal autopsy, demographic surveillance systems, population censuses, population-based household surveys and reproductive-age mortality studies (RAMOS) [7], [13]. One of the most important data collection strategies is household surveys, which can use both direct and indirect methods to measure maternal mortality. In this paper we focus on household survey data on survival of siblings.

### Household Surveys: The Sisterhood Method (Indirect and Direct)

Sibling survival histories incorporated into household surveys provide the opportunity to capture maternal deaths in developing countries where civil registration is incomplete or non-existent. In 1989, Graham et al. proposed an indirect sisterhood method for estimating maternal mortality consisting of a summary sibling history in which the respondent is asked about the number of sisters of the same mother who survived to adulthood and the number of those who have subsequently died [14]. Additional questions about the timing of death of sisters of reproductive age relative to pregnancy are used to identify pregnancy-related deaths. This method reduces the need for large samples (because in high fertility populations each respondent reports multiple sisters), but only provides estimates of mortality that reflect average experience over a lengthy period of time preceding the survey.

A direct sisterhood method based on full sibling histories proposed by Rutenberg and Sullivan has been widely applied by the Demographic and Health Survey (DHS) program [15] to provide periodic estimates of maternal mortality [16]. Respondents are asked the name and sex of each sibling born by the same mother, whether each sibling is still alive, current age if the sibling is alive, and the age at death and year of death if the sibling has died; additional questions are asked concerning deaths of sisters of reproductive age about the time of death relative to pregnancy in order to identify pregnancy-related deaths. In contrast to the indirect method, these detailed data allow adult and maternal mortality rates to be estimated for calendar-year periods and make fewer assumptions to obtain the mortality estimates. However, as these deaths are relatively rare, confidence intervals are wide and thus results are typically presented for periods of seven or ten years prior to the survey.

### Can Full Sibling Histories from Men be a Reliable Source of Maternal Mortality Data?

Sibling histories to estimate maternal mortality are usually collected in DHS’s from women of reproductive age. Sibling history information was collected from women on the basis of qualitative studies of maternal deaths suggesting that women provide more reliable data about their sisters than do men [14]. Sisters are expected to remain in contact with each other, ensuring their awareness of each other’s pregnancy and survival status. However, the DHS has also collected data from men of reproductive age in a number of surveys. This provides the opportunity to examine the assumption that sisters provide better quality maternal mortality data than brothers.

The objectives of this study are the following: 1) to investigate the use of full sibling histories collected by household surveys and reported by men to estimate all cause and maternal mortality and 2) to compare these results with all cause and maternal mortality estimates obtained from full sibling histories reported by women. Given the wide confidence intervals around estimates of maternal mortality from sibling histories collected from women, it is hoped that the use of sibling histories reported by men will improve the precision of survey-based estimates.

## Methods

### Data

This study used data from DHS surveys, which are nationally-representative household surveys that collect information on a variety of demographic and health topics. In all surveys, the DHS collects data from a sample of households, using a household listing to identify eligible women for a core women’s questionnaire [17]. In a subset of surveys, a core men’s questionnaire has been included for a subsample of the households. In one hundred and three surveys to date, the DHS has included in the women’s questionnaire a maternal mortality module collecting a full sibling history as described above. Ten surveys from nine countries added the maternal mortality module to both the women’s and men’s questionnaires; it is these surveys that were the focus of this analysis. The DHS for Nigeria (1999 survey) was excluded from the analysis because the data are reputedly not of good quality and the DHS for Eritrea (1995 survey) is a restricted data set. Using this information, we calculated adult mortality rates and maternal mortality rates using the direct method.

The DHS sibling history files record each respondent as an observation, and siblings are recorded as part of that observation. To facilitate the analysis, we restructured the data in two steps into panel data (person years), in which each sibling is counted as an observation for each year they are alive and as another observation for their year of death, if they died. Dead siblings were assumed to be exposed to the risk of dying for 6 months in their year of death. We excluded siblings with missing data on survival from the analysis, since the amount of missing data on survival was small (on average 0.3%). Sex was randomly assigned to siblings missing such data. DHS collects data in the form of current ages of living siblings and the ages at death and years since death (years of death) of dead siblings. If such information is complete, DHS calculates a date of birth for each sibling and a date of death for each dead sibling. In cases where information is missing, DHS routinely imputes missing data for dates of birth and dates of death [18]. For sisters with missing information on maternal status at death, we recoded as maternal a proportion of the deaths using age-specific proportions of maternal deaths among cases with information. We used bootstrapping to estimate the standard errors and 95% confidence intervals for all the mortality estimates.

In order to assess the sensitivity of the maternal mortality estimates with respect to missing maternal status, we also calculated maternal mortality estimates under two boundary scenarios: one under which all sister deaths with no information on maternal status were recorded as maternal and the other under which all such deaths were recorded as non-maternal deaths.

### Estimating Adult Mortality

We estimated adult age-specific mortality rates for a period of five calendar years preceding the survey. The numerators of these rates consisted of the deaths of the siblings of respondents, and the denominators consisted of person-years of exposure for the siblings of respondents. Following standard DHS methodology, survey respondents themselves were not included in the analysis of same-sex mortality (omitted from the denominator) to produce what we will call the standard DHS estimator (see the *Selection bias and weights* section below for further discussion and treatment of opposite-sex mortality) [19].

We converted age-specific mortality rates into probabilities of dying by specific ages. The corresponding survivorship ratios were then chained together to give a summary mortality measure, the probability of dying between the ages of 15 and 50 (_35_q_15_) [20]. Observations up to the last full calendar year before the survey were included. We compared estimates of male and female mortality generated from full sibling histories obtained from men with those generated from histories obtained from women.

### Estimating Maternal Mortality

We calculated several indicators of maternal mortality: the maternal mortality rate (MMRate) which is the number of maternal deaths per 1,000 women of reproductive age, the proportion of maternal deaths among deaths of females of reproductive age (PMDF) and the maternal mortality ratio (MMRatio) which is the number of maternal deaths per 100,000 live births and reflects obstetric risk. The MMRate was calculated directly from the panel data in a manner similar to adult mortality for five calendar years preceding the survey. The MMRate was derived by age standardizing the maternal mortality rates using the household age distribution of women in the household listing. The PMDF was calculated as the number of pregnancy-related deaths divided by the total deaths among women aged 15–49 and age standardized using the age distribution of women. The MMRatio was calculated by dividing the MMRate by the General Fertility Rate (GFR). The GFR is the annual number of births per 1,000 women aged 15–49 and is calculated using data from birth histories. Comparable maternal mortality indicators were calculated from full sibling histories obtained from men and those obtained from women.

Finally we pooled full sibling history data obtained from men and women to calculate female _35_q_15_ and the MMRatio. To illustrate the possible gains in efficiency from pooling the data, we calculated the percent decrease in the width of the confidence intervals using pooled data versus using the data obtained from women only. Since standard errors depend on the measurement units and may vary in the means around which they occur, the coefficient of variation is another way to determine the gains in efficiency from pooling data. We estimated the coefficient of variation (the ratio of the standard error to the mean) to compare the dispersion of adult and maternal mortality using pooled data versus data obtained from women only. A coefficient of variation of less than 10% is generally considered an acceptable level of random variation for an estimate [21], [22].

### Selection Bias and Weights

Mortality estimates obtained from sibling histories are expected to suffer from selection bias since mortality is expected to cluster within sibships. Selection bias may lead to underestimation of mortality since fewer members of high-mortality sibships (compared with low-mortality sibships of the same size) survive to appear in a survey as respondents and to report on their siblings. The most clear-cut example of this bias is sibships of which no member survives to be a potential respondent (zero-survivor bias). The standard DHS approach to analyzing sibling history data excludes the respondent from the analysis and weights only according to the respondent’s sample weight. Trussell and Rodriguez have shown mathematically that estimates obtained from the standard approach are unbiased when there are no differentials in mortality by sibship size in the data [19]. Masquelier has demonstrated that the correlation between mortality and sibship size is small for adult siblings in DHS data [23].

Expecting that mortality may vary by sibship size, Gakidou and King proposed using weights (the inverse of the number of surviving siblings of the respondent) to recover the death rates of sibships with at least one surviving respondent. They also proposed extrapolating from a model to recover deaths of sibships with no surviving members [24]. Using DHS sibling histories, a recent application of the Gakidou and King method by Obermeyer et al. adjusted adult mortality estimates for selection and omission bias [25]. Their mortality estimates were considerably higher than previously reported by studies using sibling history data as adjusting for selection bias increased estimates by approximately 27% [25]. Masquelier argues that the reported bias was overestimated due to the incorrect application of the weights to the survey data, and casts doubt on the view that mortality of adults varies substantially by sibship size; he recommends using the DHS standard approach, which does not use weights to correct for survival selection bias [23].

In the absence of differential mortality by sibship size, using the standard approach provides unbiased estimates of mortality and precludes the need to adjust for selection bias. However, analyzing reports by siblings of the opposite sex is a different matter. The respondents in this case are not exposed to the risk of dying that is being measured and there is no equivalent of the unbiased DHS standard estimator. Thus, opposite-sex sibling reports should be weighted by the inverse of the number of surviving siblings of the respondent as proposed by Gakidou and King to adjust for selection bias. Analyzing opposite-sex sibling reports has an additional advantage that sibships that have no surviving siblings of the opposite sex can be reported; all that is needed is to assume that siblings of one sex in sibships with no survivors of the other sex have mortality similar to the population average.

In this analysis, we use the DHS standard estimator (excluding respondents from the analysis) to estimate same-sex sibling mortality, thus avoiding the need to adjust for selection bias. We weighted women’s sisters and men’s brothers using the DHS sample weights only. We compared these standard estimates with adjusted estimates (obtained by using the product of DHS sample weights and the inverse of the number of surviving siblings (of the same sex) of the respondent) to isolate the effects of weighting for survival bias. The standard estimates are expected to be slightly higher than the adjusted estimates because the latter do not include deaths from sibships in which no potential respondents survive. For opposite-sex sibling mortality, we weighted women’s brothers and men’s sisters using the product of DHS sample weights and the inverse of the number of surviving siblings (of the same sex) of the respondent to adjust for selection bias. Given that DHS sibling history data are collected from women aged (15–49) and men aged 15–59 (or in some surveys aged 15–54), the surviving siblings used were of those ages in order to represent potential respondents.

### Data Quality Investigation

We assessed the completeness and accuracy of sibling history data using several indicators of data quality. We examined the completeness of the information in the histories including sex, survival status, age if alive, and, if dead, age at death and years since death. The distortion of age reporting was assessed using a modified Whipple’s Index, an index of age attraction for digits 0 and 5. The index as implemented here is the ratio of the sum of the populations aged 15, 20, 25, 30, 35, 40, 45 and 50 divided by the sum of the population aged 13 to 52 and the result is multiplied by 500. Whipple’s Index is 100 if there is no age heaping at ages ending in the digits 0 or 5. The suggested interpretation of the original Whipple’s Index is as follows: <105 is “highly accurate”; 105–109.9 is “fairly accurate”; 110–124.9 is “approximate”; 125–174.9 is “rough” and ≥175 is “very rough” [26]. The quality of the data obtained from men was compared with that obtained from women on the basis of this index.

## Results

### Adult and Maternal Mortality

**Figure 1:**
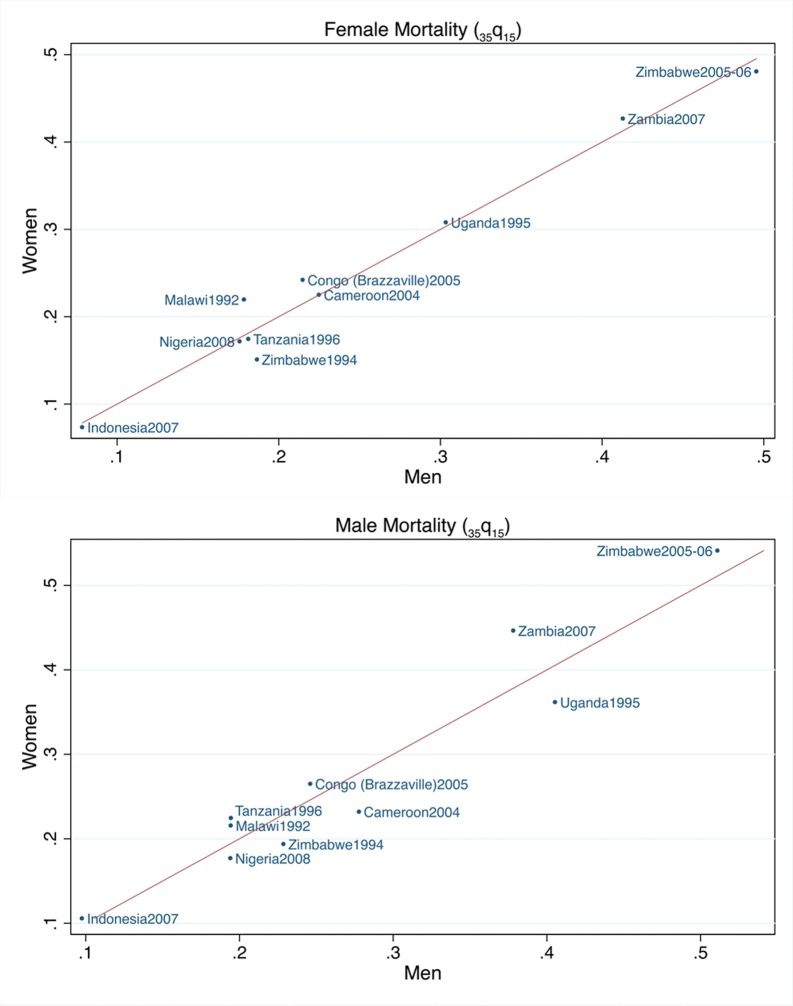
Comparison of the female and male probabilities of dying (_35_q_15_) obtained from sibling histories reported by women and men for the 1–5 calendar years preceding the survey.

The estimates and uncertainty intervals for adult mortality (the probability of dying between 15 and 50, _35_q_15_) obtained from sibling histories from women and men for the 1–5 calendar years before the surveys are listed in Table 1. The total number of respondents (women and men) and their reported siblings (living and deceased) for each survey are displayed in Table S1. In Figure 1, the measures of male and female adult mortality obtained from the female respondents are compared with those obtained from male respondents. Zambia, Zimbabwe (2005–06 survey) and Uganda showed the highest _35_q_15_’s followed by all the other African countries, while Indonesia showed the lowest values. The female mortality estimates reported by the women were higher than those reported by the men in four surveys, but the differences were not statistically significant. The male mortality estimates reported by women were higher than those reported by men in six surveys, but again the differences were not statistically significant. In Zimbabwe (the only country with two surveys that included a male questionnaire with the maternal mortality module), there was a 3- to 4- fold increase in adult mortality estimated from the 1994 survey to the 2005–06 survey. The confidence intervals did not overlap indicating a statistically significant trend.

**Table 1:** Female and male probabilities of dying (_35_q_15_) for the 1–5 calendar years preceding the survey obtained from sibling histories reported by women and men.

Female Mortality									
Country	Year of survey	Based on reports of women				Based on reports of men			
		Respondents	_35_q_15_	95% Confidence Intervals		Respondents	_35_q_15_	95% Confidence Intervals	
Cameroon	2004	10,656	0.225	0.203	0.247	5,280	0.225	0.192	0.258
Congo	2005	7,051	0.242	0.212	0.272	3,146	0.215	0.171	0.258
Indonesia	2007	32,895	0.074	0.062	0.085	8,758	0.078	0.061	0.096
Malawi	1992	8,120	0.220	0.181	0.258	2,256	0.178	0.121	0.236
Nigeria	2008	33,385	0.172	0.159	0.185	15,486	0.176	0.156	0.195
Tanzania	1996	4,849	0.174	0.146	0.203	1,151	0.181	0.134	0.229
Uganda	1995	7,070	0.308	0.273	0.343	1,996	0.303	0.243	0.364
Zambia	2007	7,146	0.427	0.390	0.464	6,500	0.413	0.377	0.449
Zimbabwe	1994	6,128	0.151	0.131	0.171	2,141	0.187	0.126	0.247
Zimbabwe	2005–06	8,907	0.481	0.457	0.504	7,175	0.495	0.452	0.538
**Male Mortality**									
**Country**	**Year of survey**	**Based on reports of women**				**Based on reports of men**			
		**Respondents**	**_35_q_15_**	**95% Confidence Intervals**		**Respondents**	**_35_q_15_**	**95% Confidence Intervals**	
Cameroon	2004	10,656	0.232	0.206	0.258	5,280	0.278	0.242	0.313
Congo	2005	7,051	0.265	0.227	0.302	3,146	0.246	0.205	0.287
Indonesia	2007	32,895	0.106	0.091	0.121	8,758	0.098	0.077	0.118
Malawi	1992	8,120	0.216	0.171	0.260	2,256	0.194	0.139	0.250
Nigeria	2008	33,385	0.177	0.161	0.193	15,486	0.194	0.176	0.212
Tanzania	1996	4,849	0.224	0.187	0.262	1,151	0.194	0.140	0.248
Uganda	1995	7,070	0.362	0.320	0.403	1,996	0.405	0.337	0.474
Zambia	2007	7,146	0.446	0.406	0.486	6,500	0.378	0.347	0.409
Zimbabwe	1994	6,128	0.194	0.166	0.221	2,141	0.229	0.184	0.273
Zimbabwe	2005–06	8,907	0.541	0.508	0.574	7,175	0.511	0.481	0.541

The female and male mortality estimates obtained from same sex respondents were adjusted for selection bias as described above and compared to the DHS standard estimates (Table S2); adjustment resulted in estimates lower than the standard approach as we anticipated. The mean of the ratios of adjusted to standard estimates of _35_q_15_ is 0.88 for both female mortality reported by women (range 0.82–0.97) and 0.88 for male mortality reported by men (range 0.74–0.99).

**Figure 2:**
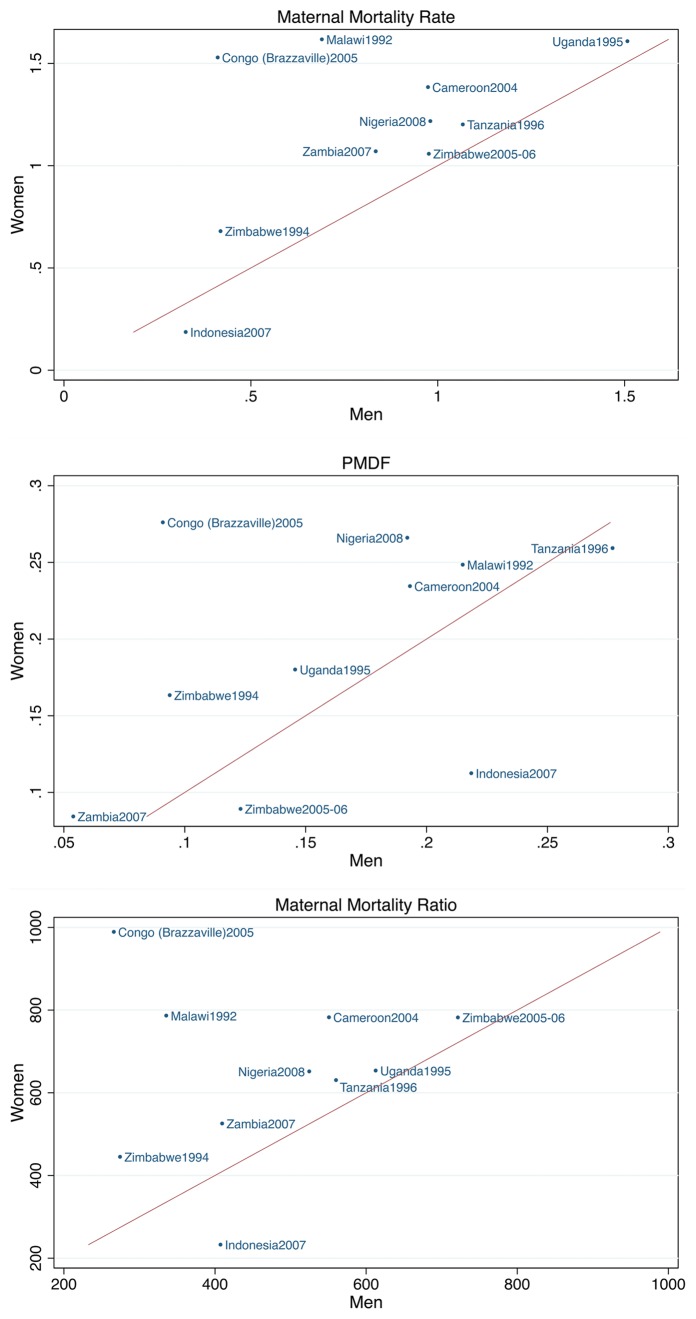
Comparison of the maternal mortality rates (MMRates), the proportions of maternal deaths among deaths of females of reproductive age (PMDFs) and the maternal mortality ratios (MMRatios) obtained from sibling histories reported by women and men for the 1–5 calendar years preceding the survey.

The measures of maternal mortality depend not only on reported survival of siblings but also on whether a reported sister death was maternal. The estimated MMRates, MMRatios and PMDFs (and their uncertainty intervals) for the 1–5 calendar years before the surveys are presented in Table 2 and Figure 2. Indonesia and Zimbabwe (1994 survey) showed the lowest MMRates. In Zimbabwe there was a 2- to 3- fold increase from the 1994 survey to the 2005–06 survey in the MMRate and MMRatio. The confidence intervals for the MMRatio reported by men did not overlap indicating a statistically significant trend. The MMRates and MMRatios based on reports of women were higher than the estimates based on reports of men in eight surveys, but the differences were only significant for Congo (Brazzaville). The PMDFs reported by women were higher than those reported by men in seven surveys, but the differences were only significant for Congo (Brazzaville) and Nigeria.

**Table 2:** Age-standardized maternal mortality rates (MMRates), proportions of maternal deaths among deaths of females of reproductive age (PMDFs) and maternal mortality ratios (MMRatios) obtained from sibling histories reported by women and men for the 1–5 calendar years preceding the survey.

Maternal Mortality Ratio							
Country	Year of survey	Based on reports of women			Based on reports of men		
		MMRate	95% Confidence Intervals		MMRate	95% Confidence Intervals	
Cameroon	2004	1.38	1.09	1.68	0.97	0.63	1.32
Congo*	2005	1.53	0.85	2.21	0.41	0.11	0.72
Indonesia	2007	0.19	0.13	0.24	0.33	0.08	0.57
Malawi	1992	1.62	0.99	2.24	0.69	0.00	1.39
Nigeria	2008	1.22	1.06	1.38	0.98	0.75	1.21
Tanzania	1996	1.20	0.89	1.52	1.07	0.54	1.59
Uganda	1995	1.61	1.19	2.03	1.51	0.72	2.30
Zambia	2007	1.07	0.75	1.39	0.83	0.48	1.19
Zimbabwe	1994	0.68	0.46	0.90	0.42	0.06	0.78
Zimbabwe	2005–06	1.06	0.79	1.32	0.98	0.63	1.32
**PMDF**							
**Country**	**Year of survey**	**Based on reports of women**			**Based on reports of men**		
		**PMDF**	**95% Confidence Intervals**		**PMDF**	**95% Confidence Intervals**	
Cameroon	2004	0.234	0.188	0.281	0.193	0.117	0.269
Congo*	2005	0.276	0.173	0.379	0.091	0.010	0.172
Indonesia	2007	0.112	0.069	0.156	0.219	0.071	0.366
Malawi	1992	0.248	0.164	0.333	0.215	0.031	0.399
Nigeria*	2008	0.266	0.234	0.298	0.192	0.151	0.233
Tanzania	1996	0.259	0.203	0.316	0.277	0.139	0.415
Uganda	1995	0.180	0.137	0.223	0.146	0.074	0.217
Zambia	2007	0.084	0.051	0.117	0.054	0.030	0.078
Zimbabwe	1994	0.163	0.111	0.216	0.094	0.000	0.196
Zimbabwe	2005–06	0.089	0.055	0.123	0.123	0.070	0.176
**Maternal Mortality Ratio**							
**Country**	**Year of survey**	**Based on reports of women**			**Based on reports of men**		
		**MMRatio**	**95% Confidence Intervals**		**MMRatio**	**95% Confidence Intervals**	
Cameroon	2004	783	616	949	551	357	745
Congo*	2005	989	551	1427	266	69	463
Indonesia	2007	233	164	301	407	102	712
Malawi	1992	786	483	1089	336	0	678
Nigeria	2008	651	565	738	525	401	648
Tanzania	1996	631	465	796	560	286	834
Uganda	1995	653	483	824	613	291	935
Zambia	2007	525	366	684	410	237	583
Zimbabwe	1994	445	299	590	274	37	511
Zimbabwe	2005–06	782	586	978	722	465	978

* Significantly different (95% level) estimates (confidence intervals do not overlap).

Female _35_q_15_ and MMRatios and corresponding uncertainty intervals calculated from pooled data and from data obtained from women only are shown in Table 3. For all surveys, pooling of data reduced the standard errors and consequently narrowed the 95% confidence intervals by 2 to 29% for female mortality and 0 to 6% for the MMRatio. Pooling data also reduced coefficients of variation, except for Tanzania.

**Table 3:** Female probability of dying (_35_q_15_) and the maternal mortality ratio (and their coefficients of variation) obtained from sibling histories data reported by women only and pooled data reported by women and men for the 1–5 calendar years preceding the survey^1^.

Female mortality										
Country	Year of survey	Based on reports of women				Based on reports of women & men				% Decrease in CI width
		35q15	95% confidence interval		Coefficient of variation	35q15	95% confidence interval		Coefficient of variation	
Cameroon	2004	0.212	0.190	0.234	5.3	0.216	0.197	0.236	4.6	12
Congo	2005	0.252	0.216	0.288	7.3	0.242	0.211	0.273	6.5	15
Indonesia	2007	0.079	0.066	0.092	8.2	0.079	0.068	0.090	7.3	11
Malawi	1992	0.221	0.180	0.263	9.5	0.213	0.176	0.251	8.9	9
Nigeria	2008	0.167	0.153	0.180	4.1	0.169	0.157	0.181	3.5	14
Tanzania	1996	0.178	0.147	0.210	9.0	0.179	0.151	0.207	8.0	11
Uganda	1995	0.279	0.242	0.315	6.7	0.282	0.252	0.313	5.5	16
Zambia	2007	0.454	0.406	0.502	5.4	0.437	0.404	0.471	4.0	29
Zimbabwe	1994	0.145	0.123	0.168	7.9	0.155	0.133	0.178	7.5	2
Zimbabwe	2005–06	0.465	0.438	0.492	3.0	0.477	0.453	0.502	2.5	8
**Maternal Mortality Ratio**										
**Country**	**Year of survey**	**Based on reports of women**				**Based on reports of women & men** 2				**% Decrease in CI width**
		**MMRatio**	**95% confidence interval**		**Coefficient of variation**	**MMRatio**	**95% confidence interval**		**Coefficient of variation**	
Cameroon	2004	736	567	904	11.7	743	579	906	11.2	3
Congo	2005	944	542	1346	21.7	899	522	1275	21.4	6
Indonesia	2007	219	146	293	17.2	217	145	289	16.9	3
Malawi	1992	747	413	1081	22.8	713	398	1027	22.5	6
Nigeria	2008	709	598	819	8.0	715	608	822	7.6	3
Tanzania	1996	620	444	797	14.5	616	439	794	14.7	0
Uganda	1995	661	463	859	15.3	669	475	863	14.8	2
Zambia	2007	564	384	745	16.3	542	372	711	16.0	6
Zimbabwe	1994	435	275	594	18.7	419	266	572	18.7	4
Zimbabwe	2005–06	749	535	963	14.6	780	567	994	14.4	0

1 In order to pool data , the data obtained from women were weighted in a similar manner to data obtained from men to adjust for selection bias; however, women’s data had to be further adjusted for zero survival bias. The deaths and exposure reported by women were weighted by the inverse of the number of surviving siblings of the respondent to adjust for selection bias and further weighted by the inverse of the probability of being reported at all to adjust for zero survival bias (men provide information on women sibships with no surviving members).

2 The PMDF (obtained from women) is multiplied by female mortality (obtained from pooled data) to generate the MMRatio.

### Sensitivity Analysis

The impact of the inclusion, partial inclusion or exclusion of sister deaths with no maternal status information on the estimates of maternal mortality is displayed in Table 4. Going from one extreme of treating all sister deaths with missing information as non-maternal to the other extreme of treating them all as maternal resulted in an increase in the estimates of maternal mortality ranging from 55 to 307% (mean 129%) for MMRate and MMRatio reported by men; 37 to 197% (mean 92%) for MMRate and MMRatio reported by women; 58 to 168% (mean 142%) for PMDF reported by men and 36 to 177% (mean 90%) for PMDF reported by women.

**Table 4:** Sensitivity analysis for maternal mortality estimates (maternal mortality rate (MMRate), proportion of maternal deaths among deaths of females of reproductive age (PMDF) and maternal mortality ratio (MMRatio)).

Country	Year of survey	Sister deaths	Based on reports of men			Based on reports of women		
			MMRate	PMDF	MMRatio	MMRate	PMDF	MMRatio
Cameroon	2004	Not included	0.86	0.169	474	1.23	0.208	679
		Proportion included	0.97	0.193	551	1.38	0.234	783
		All included	1.72	0.311	949	2.01	0.336	1109
Congo	2005	Not included	0.39	0.086	256	1.35	0.240	884
		Proportion included	0.41	0.091	266	1.53	0.276	989
		All included	0.70	0.145	457	2.12	0.375	1387
Indonesia	2007	Not included	0.27	0.175	286	0.16	0.095	171
		Proportion included	0.33	0.219	407	0.19	0.112	233
		All included	0.73	0.394	779	0.47	0.265	507
Malawi	1992	Not included	0.58	0.200	264	1.35	0.209	612
		Proportion included	0.69	0.215	336	1.62	0.248	786
		All included	2.37	0.467	1075	2.60	0.394	1182
Nigeria	2008	Not included	0.82	0.158	418	1.03	0.222	522
		Proportion included	0.98	0.192	525	1.22	0.266	565
		All included	2.02	0.396	1026	1.92	0.417	977
Tanzania	1996	Not included	0.99	0.252	491	1.10	0.238	550
		Proportion included	1.07	0.277	560	1.20	0.259	631
		All included	1.82	0.399	905	1.52	0.324	756
Uganda	1995	Not included	1.38	0.133	559	1.40	0.157	569
		Proportion included	1.51	0.146	613	1.61	0.180	653
		All included	2.14	0.222	868	2.74	0.302	1110
Zambia	2007	Not included	0.79	0.051	392	1.00	0.077	496
		Proportion included	0.83	0.054	410	1.07	0.084	525
		All included	1.65	0.136	814	1.95	0.185	964
Zimbabwe	1994	Not included	0.38	0.083	229	0.63	0.152	379
		Proportion included	0.42	0.094	274	0.68	0.163	445
		All included	0.72	0.177	434	0.99	0.230	599
Zimbabwe	2005–06	Not included	0.92	0.118	661	0.97	0.082	697
		Proportion included	0.98	0.123	722	1.06	0.089	782
		All included	2.28	0.187	1636	2.28	0.170	1638

### Data Quality Investigation

The completeness of the sibling history data in all the surveys is displayed in Table 5. With regard to the completeness of sibling history information, on average for women and men respectively 0.23% (range 0.05–0.62%) and 0.35% (range 0.04–1.55%) of data were missing on survival status, 0.31% (range 0.04–0.89%) and 0.36% (0.01–1.16%) of data were missing on sex and 1.56% (range 0.14–2.28%) and 1.80% (range 0.08–4.82%) of data were missing for age of living siblings. For the age at death and year of death (or years since death) of the dead siblings, 1.60% (range 0.14–4.00%) was missing for women and 1.80% (range 0.08–4.82%) was missing for men. For the maternal status information, 11% (range 6–19%) and 12% (range 7–22%) were missing, respectively; the percentage of deaths of reproductive age females with no information on their maternal mortality status reported by both sexes was largest for Indonesia, Tanzania and Uganda. The data obtained from women were in general slightly more complete on average than the data obtained from men.

**Table 5:** Completeness of sibling history information on survival status, current age if alive, age at death and years since death if dead and maternal mortality status.

Country	Year of survey	Alive/Dead Unknown		Current age Unknown		AD & YSD* Unknown		Maternal status Unknown	
		(Percent of total siblings)		(Percent of total living siblings)		(Percent of deceased siblings)		(Percent of deceased sisters ages 15–49)	
		Women	Men	Women	Men	Women	Men	Women	Men
Cameroon	2004	0.05	0.04	0.79	0.62	1.32	1.87	8.92	8.49
Congo	2005	0.09	0.10	0.34	0.60	1.58	2.44	9.14	7.06
Indonesia	2007	0.06	0.23	0.74	0.44	1.98	2.94	15.44	17.77
Malawi	1992	0.58	0.31	0.07	0.06	0.26	0.08	15.43	20.38
Nigeria	2008	0.62	0.59	0.98	1.09	2.11	2.77	18.77	22.20
Tanzania	1996	0.10	0.17	0.61	0.48	2.28	0.70	7.17	14.45
Uganda	1995	0.44	1.55	1.37	1.08	4.00	4.82	13.81	7.15
Zambia	2007	0.17	0.17	0.52	0.65	1.25	1.01	5.78	7.18
Zimbabwe	1994	0.09	0.17	0.17	0.07	0.14	0.63	8.71	10.51
Zimbabwe	2005–06	0.12	0.15	0.74	0.59	0.71	0.75	11.11	8.08
Average		0.23	0.35	0.63	0.57	1.56	1.80	11.43	12.33

* Age at death (AD) & Years since death (YSD).

In general, age heaping of the siblings on ages ending in digits 0 and 5 (both living and dead) was in the “approximate” category and ranged from “highly accurate” to “rough” (Table 6). Overall, men appeared to report their siblings’ ages with less distortion than women, especially their brothers’ ages at death. Women reported their sisters’ ages at death with less distortion than their brothers’ ages at death in the majority of surveys, whereas the men reported their sisters’ ages at death with less distortion in some surveys and their brothers’ ages at death with less distortion in others.

**Table 6:** Age reporting distortion in sibling histories obtained from women and men.

Country	Year of survey	Modified Whipple’s Index* of current ages of living sibling				Modified Whipple’s Index* of ages at death of deceased sibilngs			
		Sister		Brother		Sister		Brother	
		Women	Men	Women	Men	Women	Men	Women	Men
Cameroon	2004	122	114	120	113	138	150	167	155
Congo	2005	115	116	121	115	128	121	161	131
Indonesia	2007	123	132	129	120	200	186	208	177
Malawi	1992	107	110	106	105	141	138	145	99
Nigeria	2008	151	133	138	155	186	167	184	166
Tanzania	1996	119	113	116	116	137	126	140	138
Uganda	1995	122	122	116	125	145	155	151	138
Zambia	2007	111	112	111	111	118	120	133	131
Zimbabwe	1994	113	109	111	106	139	122	142	109
Zimbabwe	2005–06	108	112	121	113	121	123	137	121
Average		119	117	119	118	145	141	157	136

* Modified Whipple’s Index: an index of age attraction for digits 0 and 5. The index is the ratio of the sum of the populations aged 15, 20, 25, 30, 35, 40, 45 and 50 divided by the sum of the population aged 13 to 52 and the result is multiplied by 500. Suggested interpretation is as follows: <105 is “highly accurate”; 105–109.9 is “fairly accurate”; 110–124.9 is “approximate”; 125–174.9 is “rough” and ≥175 is “very rough”.

## Discussion

A major challenge to estimating adult mortality and maternal mortality in particular, is that estimates obtained from surveys have large standard errors and thus are not useful for monitoring trends. The findings of this study suggest that efficiency gains in estimating adult and maternal mortality can be obtained from collecting male sibling histories in addition to female sibling histories. This provides a way for countries to generate robust empirical estimates of adult and maternal mortality from surveys, which in turn improves the accuracy of tracking progress towards MDG-5 and monitoring trends in adult and maternal mortality.

Our analysis shows that adult and maternal mortality estimates based on sibling histories obtained from men do not systematically vary from those obtained from women. The exception to this was the Congo (Brazzaville) DHS, in which men reported significantly lower maternal mortality estimates than women, although both sexes reported similar all-cause sister mortality indicating comparable reporting of sister survival but differing reporting of pregnancy status at death. Several studies have documented an increase in maternal mortality during times of war and conflict [27], [28]. A civil war occurred in Congo between 1997 and 1999, and it may be that social dislocation might have led male respondents to be aware of their sisters’ survival status but not their pregnancy status at death in time of war. Women are also at an elevated risk of experiencing sexual violence and rape and consequently pregnancy during times of conflict [29].

The sibling history data quality indicators were similar for women and men in this study. We observed that brothers generally provide slightly better quality information on their siblings’ current ages and ages at death. Given the similarity in mortality estimates and data quality indicators between women and men, there is no reason to believe that male respondents do not provide estimates of sister mortality as reliable as those from female respondents. In addition, pooling sibling history data obtained from men with data obtained from women increases sample size and produces narrower confidence intervals and lower coefficients of variation, although for most countries the MMRatio coefficients of variation remained higher than 10%. The exception to this was Tanzania, where pooling data produced a slightly higher coefficient of variation, which could be explained by a higher level of random variation in male reports. The sample size advantage would be maximized by collecting sibling history data from male and female respondents in all sampled households rather than following the DHS practice of interviewing males in only a subsample of households.

The DHS Interview Manual states that an interviewer’s role involves “Interviewing all eligible respondents in the households using the individual Woman’s or Man’s Questionnaire” [30]. Therefore, the cost of including a full sibling history in the male questionnaire, if men are interviewed in all sampled households, is the incremental cost of asking the sibling history questions. However, if men were only interviewed in a subsample of households, then the cost would be the incremental cost of interviewing an additional household member. Unfortunately, detailed data on the cost and time of DHS interviews are not available to estimate the specific additional time and cost required.

Given the rarity of maternal deaths, the omission or addition of a few cases can lead to disproportionate effects on the maternal mortality estimates. These effects are reflected in the sensitivity analyses, which demonstrated the impact that the completeness and method of imputation of maternal status data has on DHS estimates of maternal mortality. The whole debate around survival selection bias and its effect on mortality is dwarfed by the influence of the completeness of maternal status data on the estimates, and additional effort is needed during training of interviewers to ensure omission is kept to a minimum.

The majority of the surveys in this study were conducted in African countries, where the effect of HIV/AIDS on adult mortality is clear, especially in southern African countries like Zambia and Zimbabwe. HIV/AIDS has the potential to affect our estimates of mortality to the extent that there is clustering of HIV and HIV mortality among adult siblings. This clustering might lead to downward bias in retrospectively-reported deaths because only surviving siblings are able to report (see the *Selection bias and weights* section above). In high HIV prevalence settings, methods of estimation of child mortality are subject to downward bias due to the correlation between HIV-related mortality of mothers and their children [31], [32]. However, HIV correlation among siblings and its potential effect on estimates of mortality obtained from sibling histories is not well established. In our analysis, we eliminated the need to adjust for selection bias in same-sex sibling mortality estimates by using the DHS standard estimator and we adjusted for potential selection bias in opposite-sex sibling mortality estimates by using weights.

This study has several limitations. Sibling histories were mainly included in women’s questionnaires, and the number of men’s questionnaires that incorporated a maternal mortality module was thus limited, with the majority being from African countries. The use of sibling histories to estimate adult mortality also has its limitations, including a limited number of events, survival selection bias and the omission of deaths. In this study, we assumed in the analysis of same-sex mortality that there was no correlation between mortality and sibship size, which eliminates the need to adjust for survival selection bias; we did however use weights based on numbers of survivors for opposite-sex mortality. For all countries except one, only one survey was available, which precluded our ability to adjust for omission bias.

## Conclusion

Sibling histories have been collected from women in household surveys with the aim of estimating maternal mortality but have not been widely collected from men due to concerns about data quality. This study has found that male and female respondents report sibling histories that provide similar adult mortality estimates, maternal mortality estimates (except for Congo Brazzaville) and data quality indicators. Given that no significant differences are found between adult and maternal mortality estimates obtained from women and men, data can be pooled to increase precision of the estimates (narrower confidence intervals and lower coefficients of variation). We therefore advocate that sibling histories be collected from both men and women and that the histories obtained from men be collected from all sampled households.

## Supporting Information

Table S1 **Number of respondents (women and men) and number of reported siblings (living and deceased).**
(DOCX)

Table S2 **Standard and adjusted adult mortality (_35_q_15_) reported by the same sex respondents.**
(DOCX)
