# Supplementary material for: Improving the Measurement of Maternal Mortality: The Sisterhood Method Revisited
Source: PLoS One. 2013 Apr 2;8(4):e59834. doi: 10.1371/journal.pone.0059834 (PMC3614991; doi:10.1371/journal.pone.0059834)
Supplement: Table S1 — Number of respondents (women and men) and number of reported siblings (living and deceased). (DOCX) [file pone.0059834.s001.docx]

# Supporting Information

Table S1: Number of respondents (women and men) and number of reported siblings (living and deceased).

| Country | Year | Total Respondents | | Living Siblings | | Deceased Siblings | |
| --- | --- | --- | --- | --- | --- | --- | --- |
|  |  | Women | Men | Women | Men | Women | Men |
| Cameroon | 2004 | 10,656 | 5,280 | 51,061 | 24,267 | 14,403 | 7,583 |
| Congo (Brazzaville) | 2005 | 7,051 | 3,146 | 33,089 | 14,239 | 7,665 | 3,810 |
| Indonesia | 2007 | 32,895 | 8,758 | 126,637 | 33,630 | 17,115 | 5,460 |
| Malawi | 1992 | 4,849 | 1,151 | 20,804 | 4,933 | 9,190 | 1,890 |
| Nigeria | 2008 | 33,385 | 15,486 | 148,485 | 67,097 | 30,322 | 16,017 |
| Tanzania | 1996 | 8,120 | 2,256 | 39,228 | 10,389 | 8,539 | 2,575 |
| Uganda | 1995 | 7,070 | 1,996 | 34,314 | 9,599 | 9,621 | 2,810 |
| Zambia | 2007 | 7,146 | 6,500 | 33,476 | 30,215 | 7,695 | 7,131 |
| Zimbabwe | 1994 | 6,128 | 2,141 | 32,708 | 11,286 | 5,780 | 2,224 |
| Zimbabwe | 2005-06 | 8,907 | 7,175 | 40,198 | 31,608 | 6,897 | 5,850 |
