# Supplementary material for: Improving the Measurement of Maternal Mortality: The Sisterhood Method Revisited
Source: PLoS One. 2013 Apr 2;8(4):e59834. doi: 10.1371/journal.pone.0059834 (PMC3614991; doi:10.1371/journal.pone.0059834)
Supplement: Table S2 — Standard and adjusted adult mortality (35q15) reported by the same sex respondents. (DOCX) [file pone.0059834.s002.docx]

# Supporting Information

Table S2: Standard and adjusted adult mortality (_35_q_15_) reported by the same sex respondents.

| Female Mortality (reported by women) | | | | |  |  | |
| --- | --- | --- | --- | --- | --- | --- | --- |
| Country | Year | Standard (Non-weighted) | | | Adjusted (weighted) | | |
|  |  | _35_q_15_ | 95 % Confidence Interval | | _35_q_15_ | 95 % Confidence Interval | |
| Cameroon | 2004 | 0.225 | 0.203 | 0.247 | 0.189 | 0.169 | 0.208 |
| Congo | 2005 | 0.242 | 0.212 | 0.272 | 0.235 | 0.201 | 0.269 |
| Indonesia | 2007 | 0.074 | 0.062 | 0.085 | 0.062 | 0.052 | 0.072 |
| Malawi | 1992 | 0.220 | 0.181 | 0.258 | 0.191 | 0.154 | 0.228 |
| Nigeria | 2008 | 0.172 | 0.159 | 0.185 | 0.142 | 0.130 | 0.153 |
| Tanzania | 1996 | 0.174 | 0.146 | 0.203 | 0.154 | 0.126 | 0.182 |
| Uganda | 1995 | 0.308 | 0.273 | 0.343 | 0.253 | 0.219 | 0.287 |
| Zambia | 2007 | 0.427 | 0.390 | 0.464 | 0.410 | 0.364 | 0.455 |
| Zimbabwe | 1994 | 0.151 | 0.131 | 0.171 | 0.131 | 0.110 | 0.151 |
| Zimbabwe | 2005-06 | 0.481 | 0.457 | 0.504 | 0.435 | 0.409 | 0.462 |
| Male Mortality (reported by men) | | | | |  | |  |
| Country | Year | Standard (Non-weighted) | | | Adjusted (weighted) | | |
|  |  | _35_q_15_ | 95 % Confidence Interval | | _35_q_15_ | 95 % Confidence Interval | |
| Cameroon | 2004 | 0.278 | 0.242 | 0.313 | 0.240 | 0.207 | 0.273 |
| Congo | 2005 | 0.246 | 0.205 | 0.287 | 0.211 | 0.171 | 0.251 |
| Indonesia | 2007 | 0.098 | 0.077 | 0.118 | 0.072 | 0.057 | 0.088 |
| Malawi | 1992 | 0.194 | 0.139 | 0.250 | 0.160 | 0.107 | 0.213 |
| Nigeria | 2008 | 0.194 | 0.176 | 0.212 | 0.170 | 0.153 | 0.188 |
| Tanzania | 1996 | 0.194 | 0.140 | 0.248 | 0.175 | 0.122 | 0.228 |
| Uganda | 1995 | 0.405 | 0.337 | 0.474 | 0.345 | 0.290 | 0.400 |
| Zambia | 2007 | 0.378 | 0.347 | 0.409 | 0.348 | 0.316 | 0.380 |
| Zimbabwe | 1994 | 0.229 | 0.184 | 0.273 | 0.212 | 0.163 | 0.262 |
| Zimbabwe | 2005-06 | 0.511 | 0.481 | 0.541 | 0.51 | 0.472 | 0.549 |
